# Prevalence and associated factors of modern contraceptive discontinuation among sexually active married women in Nigeria

**DOI:** 10.1186/s40834-022-00205-9

**Published:** 2023-01-13

**Authors:** J. A. Kupoluyi, B. L. Solanke, O. M. Adetutu, J. O. Abe

**Affiliations:** Department of Demography and Social Statistics, Faculty of Social Sciences, Obafemi Awolowo University, Ile-Ife, Nigeria

**Keywords:** Modern contraceptive discontinuation, Sexually active, Married women, Nigeria

## Abstract

**Background:**

Contraceptive discontinuation for reasons other than the desire for pregnancy is associated with a high rate of unintended pregnancies leading to unsafe abortions, maternal morbidity and mortality. In Nigeria, little is known about modern contraceptive discontinuation using the calendar data.

**Methods:**

A cross-sectional research design from the 2018 Nigeria Demographic and Health Surveys (NDHS) women’s dataset was used to examine the prevalence and associated factors of modern contraceptive discontinuation among sexually active married women in Nigeria. A weighted sample size of 3,353 currently sexually active married or in union women who have ever used a modern contraceptive 5 years before the survey and with complete reproductive histories and are not sterilised or declared infecund was analysed. Data were analysed and displayed using frequency tables and charts, chi-square test, and binary logistic regression model at 5% level of significance.

**Results:**

The prevalence of modern contraceptive discontinuation was 35.8% (1199) with 45.8% (549) of the women discontinuing using modern contraceptives while at risk of pregnancy. The most modern method discontinued was Injectables (25.2%) while the commonest reason for modern method discontinuation was because they wanted to become pregnant (36.1%). Associated factors of modern contraceptive discontinuation among sexually active married women in Nigeria were: marital duration (aOR = 3.0; 95%CI: 1.5–6.2), visitation to a health facility in the last 12 months before the survey (aOR = 0.6; 95%CI: 0.4–0.8), education (aOR = 2.0; 95%CI: 1.2–3.4) and region of residence (aOR = 2.7; 95%CI: 1.6–4.7).

**Conclusion:**

Modern contraceptive discontinuation among the study respondents was high. Region of residence, health facility visitation and marital duration were significantly associated with modern contraceptive discontinuation**.** The study suggests that health care providers should address the discontinuation of contraception through counselling, particularly among women who reside in the region of high prevalence of contraceptive discontinuation, short-term users as well as strengthen the use of contraception among those who are still at risk of becoming pregnant. Governments and stakeholders should also partner with private sectors to make health care accessible to women by bring health facilities closer to them to improve facility visitation.

## Background

The interruption of contraceptive use for one month or longer by women who had used a contraceptive method in the past 12 months but discontinued at least once without switching to another method has been identified as a clog in achieving maternal and child health-related Sustainable Development Goals (SDG) [1–3]. Discontinuation of contraceptive use, while women are still at risk of becoming pregnant without switching to any other contraceptive methods, has adverse reproductive health consequences [4, 5]. It has adverse effects on family planning programmes as well as grave implications for demographic growth [4].

According to the World Health Organisation (WHO), contraceptive discontinuation for reasons other than the desire for pregnancy is associated with a high rate of unintended pregnancies leading to unsafe abortions, maternal morbidity and mortality [2, 6]. Globally, as of 2019, about 270 million women who were sexually active and wanted to avoid pregnancies and/or postponed births were not using modern contraceptives [7–9].

Every year, in developing countries, about 74 million unintended pregnancies occur particularly among a great number of women who are not using any contraceptive [8–10]. However, consistent and effective use of modern methods of contraception could prevent unintended pregnancies and deaths of women from pregnancy-related causes [10–12]. For instance, globally in 2019, about 85% of women who were not using any contraceptive method became pregnant during the first year of discontinuation of contraception [13]. Subsequently, at least one in four pregnancies was unintended in the year 2019 [14–16]. Despite these negative reproductive health consequences of not using a contraceptive method, a large number of sexually active women who intend to avoid pregnancy are not using any contraceptive method for various reasons [17, 18]. These include the fear of contraceptive side effects, method failure, menstrual disruption, husband/spousal disapproval, menopause, fear of infertility, and desire for more children [19, 20]. Extant literature has shown that 65% of sexually active women (15–49) who have an unmet need for contraception in 2018, stopped using contraception because of the fear of side effects, and health risks/concerns [2, 12]. Meanwhile, contraceptive discontinuation while still at risk of becoming pregnant has a wide range of health risks for the mother and child [21]. These include high-risk pregnancies and childbirths, unsafe abortions, and low educational and employment opportunities, among others [22–24]. The WHO stated that consistent and effective use of modern methods of contraception offers a better opportunity for couples to limit and space the number of children they want [25]. It also helps in reducing maternal morbidity and mortality [26]. Studies on maternal mortality have shown that about 44% of maternal deaths were prevented through the consistent use of contraceptives [21, 27]. In the last two decades in Nigeria, among currently married women of reproductive age (15–49), the overall use of contraception has increased from 15 to 17% [28]. Within the same period, the use of any modern method of contraception has increased by 2% (from 10 to 12%) with a noticeable rise in the use of implants and injectables from 0 to 3% [28]. Despite the overall increase in modern contraceptive use in the 5 years preceding the 2018 Demographic and Health Survey [DHS] in Nigeria, two out of every five contraceptive users (40.6%) discontinued the method within 12 months [28]. In addition to this, the proportion of women who switched to another method after discontinuation of the previous method has reduced from 4.8% in 2013 to 4.5% in 2018 [28]. This high rate of modern contraceptive discontinuation could cause Nigeria to lose the progress made in increasing contraceptive use over the past 20 years.

There have been several studies [5, 6, 29–31] on why contraceptive users decide to continue or discontinue contraception. However, successive studies on factors influencing modern contraceptive discontinuation in Nigeria using national data is limited. This study, therefore, tries to fill this gap by using the 2018 NDHS calendar data and raising two research questions. What is the prevalence of modern contraceptive method discontinuation, and what are the associated factors influencing modern contraceptive discontinuation among sexually active married women in Nigeria? The 2018 NDHS calendar data covers month-by-month information on episodes of contraceptive use for the five years preceding the survey. Thus, this study examines the prevalence and associated factors of modern contraceptive method discontinuation among sexually active married women in Nigeria. The finding will inform achieving maternal and child health-related Sustainable Development Goals (SDG) [32], and the Family Planning Blueprint (scale-up plan) in Nigeria [33] by reducing maternal morbidity and mortality as well as unintended pregnancies.

### Theoretical framework

The theory of the Health Belief Model (HBM) [34] provides theoretical support for this study. The HBM is a good framework for explaining and predicting contraceptive behaviour (i.e. probability of modern contraceptive continuation or discontinuation). The theory postulates that health-related behaviour hinges on perceived susceptibility and severity of becoming ill with a disease or health condition. Perceived benefits of preventive behaviour, perceived barriers to preventive behaviour, cues to action, and self-efficacy for preventive behaviour. The concepts of HBM as applied to modern contraceptive behaviour are to be achieved through activities involved in the process of health decision-making such as identifying and using a modern contraceptive method to prevent unwanted pregnancy. This process of health decision-making includes; Perceived susceptibility; knowledge, belief and the risk of getting pregnant). Perceived severity; beliefs about the social and medical consequences of pregnancy and childbirth. Perceived benefits; knowledge and belief about contraceptive efficacy and long-term convenience. Perceived barriers; beliefs about the side effect, risks and costs of accepting a new health behaviour. Cues to action; both internal and external stimuli such as counselling, media exposure, and missed menstrual period after intercourse could trigger the health behaviour). Self-efficacy; contraceptive decision-making and the probability of modern contraceptive continuation or discontinuation [35]. The combination of these factors together with the modifying and enabling factors (socio-demographic, psychological, structural, and reproductive) interact and triggers a reaction that repeatedly exhibits the likelihood of modern contraceptive continuation or discontinuation [36, 37]. In Nigeria, women's use of Family Planning (FP) services particularly modern contraceptives is low. Thus, HBM provides the path to understanding the factors influencing modern contraceptive behaviour and the strategies to prevent unintended pregnancy.

## Methods

### Study design and data source

The 2018 NDHS data used for this study adopted a cross-sectional research design. The women’s individual recode (IR) data extracted from the survey was used for the study. The data is accessible at https://dhsprogram.com/data/available-datasets.cfm.

### Inclusion and exclusion criteria

**Fig. 1:**
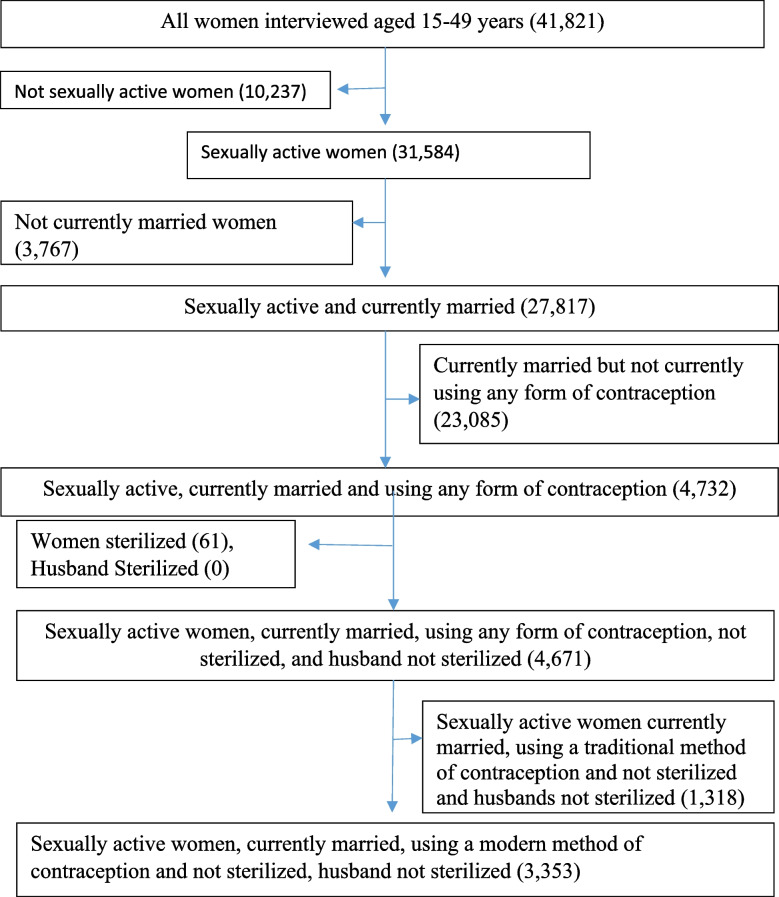
Sampling flowchart

Women who were not sexually active and not married/in-union, women who had never used contraceptives, women who had indicated that they were pregnant at the time of the survey, infecund women who were self-reported, and women with incomplete contraceptive information were excluded in this study. Also, out of the total sample of 41,821 women (15–49 years) in the IR data file, a weighted sample size of 3,433 currently sexually active married or in the union who were using a modern contraceptive and not sterilised or declared infecund were included and analysed (Fig 1).

### Handling of missing values

In DHS, the use of contraception is generally not allowed to be missing in any month in the calendar. In the few surveys where it is missing, they are treated as months of non-use of contraception. Thus, missing and unknown reasons for discontinuation are treated as “Other” reasons in this study.

### Definition and measurement of variables

#### Dependent variable

The dependent variable is the modern contraceptive discontinuation. Contraceptive discontinuation refers to the disruption of the use of modern contraceptive methods for at least 12 months before the survey. It was operationalised as a dichotomous variable, coded as '1' for currently married women who are using contraception 12 months before the survey, but discontinue before the end of the 12 months and coded as '0' otherwise. However, we are not concerned with all contraceptive discontinuation because the fertility desires of currently married women change over time. Therefore, in this analysis, discontinuation was further disaggregated based on whether discontinuation occurred even though they are still at risk of unwanted pregnancy or not. Thus, discontinuation while still at risk was coded as “1” and as ‘0’ if otherwise.

### Independent variables

The independent variables considered in this study were: age, household wealth, region, and education. Others include the history of visiting a health facility in the last 12 months, media exposure and marital duration. Age was categorised into 15–19, 20–24, 25–29, 30–34, 35–39, 40–44, and 45–49. Household wealth; women’s household ownership of assets, was classified into the poorest, poorer, middle, richer and richest. The region of residence in which respondents were interviewed was grouped into North West, North East, North Central, South West, South East and South-South. Women’s highest level of education attained was grouped into no education, primary, secondary, and higher. The history of visiting health facilities in the last 12 months was grouped as yes and no. Media exposure was defined based on the use of at least one of the news outlets. It was grouped as exposed and not exposed. Finally, marital duration was calculated by subtracting the age at the time of marriage from the age at the time of the survey, in completed years. It was categorised as 0–4, 5–9, 10–14, 15–19, 20 years or more. The variables were included in the study and used to build models based on evidence from existing literature regarding factors associated with contraceptive discontinuation and/or based on a theoretical association.

## Data analysis

Data were analysed in three stages (univariate, bivariate and multivariable) using the Stata statistical package version 14. First, frequencies and percentages were used to describe the categorical variables while the mean and standard deviation (SD) values were used for the continuous variables. Second stage, Chi-square test was used to assess bivariate associations between independent variables and contraceptive discontinuation at 5% level of significance Tests for collinearity among independent variables were examined using variance inflation factor. Using variance inflation factor > 0.5, all independence variables that were significantly associated with modern contraceptive discontinuation were moved to the logistic regression model in the third stage. Stata complex survey (svy) commands which declares data as a survey data in Stata and the women’s sample weights (v005/1000000) which also cater for stratified sample design and the effect of oversampling or under-sampling of some regions or areas were employed as recommended by DHS [18]. Finally, in the multivariable analysis, a logistic regression model at 5% level of significance was used to examine the determinants of contraceptive discontinuation up to 12 months. In this study, we focused on women who discontinued using modern contraceptive methods (pills, IUDs, injectables, implants, female condoms, and male condoms). To avoid bias as a result of the failure of some women to realise that they are pregnant in their first trimester, we censored discontinuation on the month of the interview or two months before the survey.

## Results

### Univariate analysis

Table 1 shows socio-demographic characteristics of the respondents. The mean age (SD) of the respondents was 32.4 (± 6.4) years. Above half (55%) were aged 25–34 years. Most of the respondents (44.4%) were in the richest households' quintile. About one-third of the respondents (32.3%) were from South West geo-political zone. The majority of the respondents (74.6%) had secondary school and above. On marital duration, more than half (55.6%) of the respondents had between 5–9 and 10–14 marital duration (33.9% and 21.7% respectively). About a third of the respondents (31.1%) have had media exposure to family planning messages. Almost three of every five respondents (59.3%) had visited a health facility in the last 12 months.

**Table 1:** Socio-demographic characteristics of respondents *n* = 1199

Variables	Women at risk of pregnancy		
	Total (%)	NO (%)	Yes (%)
Total	1199 (100.0)	650 (54.2)	549 (45.8)
Age (years)			
15–19	8 (0.7)	1.2	0.2
20–24	92 (7.7)	7.8	7.5
25–29	325 (27.1)	30.9	22.6
30–34	334 (27.9)	29.6	25.9
35–39	277 (23.1)	21.5	25.0
40–44	122 (10.2)	6.9	14.1
45–49	41 (3.4)	2.3	4.7
Wealth Index			
Poorest	51 (4.2)	4.3	4.1
Poorer	108 (9.0)	10.1	7.7
Middle	178 (14.8)	14.2	15.6
Richer	330 (27.5)	26.1	29.2
Richest	532 (44.4)	45.2	43.5
Region			
North Central	146 (12.2)	14.3	9.7
North East	138 (11.5)	12.8	10.0
North West	177 (14.8)	11.6	18.6
South East	185 (15.5)	17.1	13.6
South South	165 (13.8)	13.6	14.0
South West	387 (32.3)	30.6	34.2
Education			
No education	140 (11.7)	12.8	10.4
Primary	165 (13.7)	13.1	14.4
Secondary	620 (51.7)	50.4	53.2
Tertiary	274 (22.9)	23.6	21.9
Marital duration			
0–4	186 (15.5)	18.1	12.5
5–9	407 (33.9)	37.5	29.7
10–14	260 (21.7)	22.0	21.3
15–19	196 (16.3)	14.4	18.6
20 +	150 (12.5)	8.0	17.9
Media exposure			
No	826 (68.9)	70.8	66.7
Yes	372 (31.1)	29.2	33.3
Visited Health Facility in the Last 12 Months			
No	488 (40.7)	34.2	48.5
Yes	711 (59.3)	65.8	51.5

### Modern contraceptive discontinuation

**Fig. 2:**
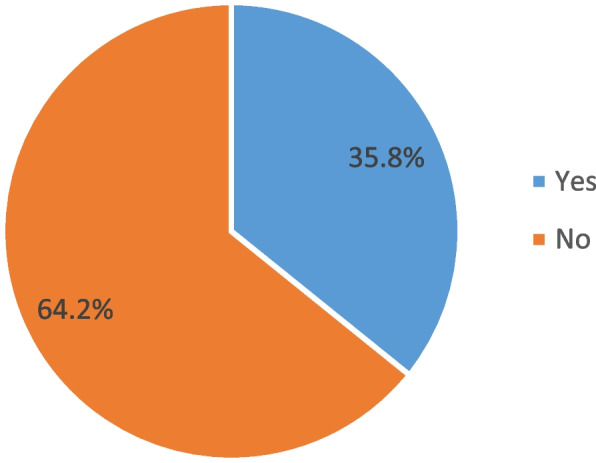
Modern contraceptive discontinution among sexually active married women in Nigeria, 2018 NDHS

Figure 2 shows the proportion of sexually active married/in-union women who met the inclusion criteria in this study. Out of 3,353 sexually active married/in union women who have ever used a modern contraceptive 5 years before the survey and with complete reproductive histories and are not sterilised or declared infecund, 35.8% (1199) discontinued contraceptives and 64.2% (2,154) did not discontinue using a modern contraceptive.

**Fig. 3:**
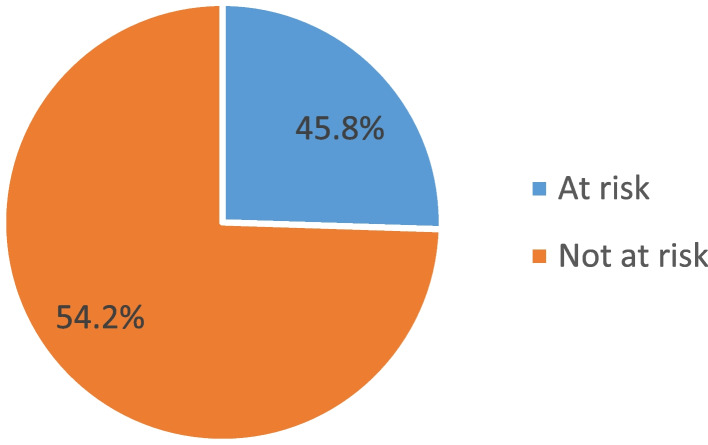
Modern contraceptive discontinution among sexually active married women at risk of pregnancy in Nigeria, 2018 NDHS

Figure 3 shows the proportion of the sexual married/in the union who have discontinued modern contraceptives during the 12-month observation period. Out of 1199 women who have discontinued using modern contraceptives, more than half (54.2%) discontinued contraceptives while not at risk of pregnancy and 45.8% discontinued contraceptives while at risk of pregnancy.

**Fig. 4:**
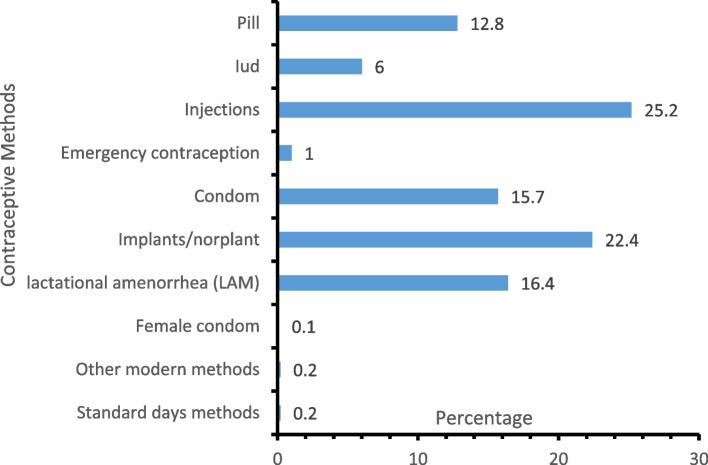
Contraceptive method discontinued at risk pregnancy by respondents

Figure 4 shows that injections (25.2%) were the most discontinued modern method. This was followed by implants/Norplant (22.4%), lactation amenorrhea (16.4%), male condoms (15.7%), pills (12.8%), and IUD (6.0%).

**Fig. 5:**
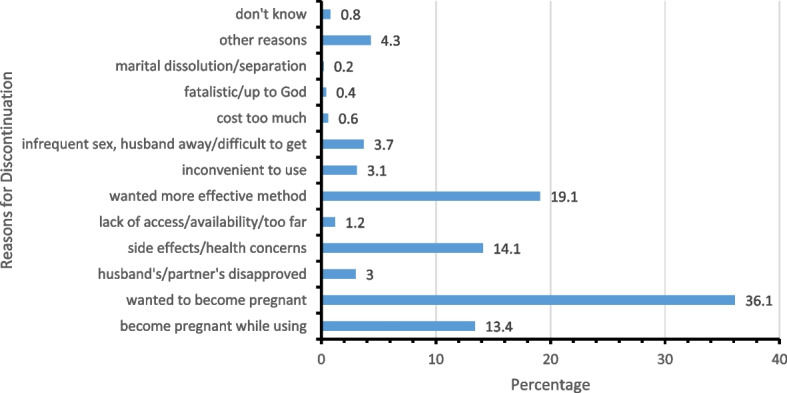
Reasons for modern contraceptive discontinuation at risk of pregnancy by respondents

The commonest reasons for modern contraceptive discontinuation (see Fig. 5) were the need to become pregnant (36.1%), followed by the need for a more effective method (19.1%), side effects/health concerns (14.1%), and became pregnant while using (13.4%).

### Bivariate and multivariable analyses

The unadjusted bivariate results in Table 2 show a statistically significant association between modern contraceptive discontinuation and the time respondents last visited a health facility. Women who visited a health facility in the last 12 months before the survey were 45% less likely to discontinue modern contraceptive methods compared to women who have not visited a health facility in the last 12 months before the survey. The data also revealed a positive association between modern contraceptive discontinuation and marital duration. Marital duration increases consistently with modern contraceptive discontinuation. That is, the higher the marital duration, the higher the odds of modern contraceptive discontinuation. This relationship is statistically significant among women whose marital duration and 20 years and above. Also, the region of residence is significantly associated with modern contraceptive discontinuation. The data revealed that women in the North West and women in the South West have a higher odds of modern contraceptive discontinuation than women in the Northern Central. Furthermore, as regards education, the results showed a significant inverse relationship between educational level and modern contraceptive discontinuation. The higher the educational level, the lower the modern contraceptive discontinuation. On the wealth quintile, the analysis showed a positive relationship between the wealth quintile and modern contraceptive discontinuation.

**Table 2:** Logistic Regression results of modern contraceptive discontinuation on associated factors

Variables	Total	Crude Odd Ratio (OR)	95% CI	Adjusted Odd Ratio (aOR)	95% CI
Visited Health Facility in the Last 12 Months					
No (RC)	488	1		1	
Yes	711	0.55*	0.41–0.75	0.58*	0.43–0.78
Media exposure					
No (RC)	826	1		1	
Yes	373	1.22	0.89–1.67	1.17	0.83–1.63
Marital duration					
0–4 (RC)	186	1		1	
5–9	407	1.14	0.73–1.76	1.13	0.70–1.82
10–14	206	1.39**	0.88–2.20	1.30	0.79–2.16
15–19	196	1.86**	1.13–3.07	1.65	0.91–2.98
20 +	150	3.23**	1.92–5.42	3.02**	1.48–6.16
Region					
North Central (RC)	146	1		1	
North East	138	1.15	0.69–1.94	1.42	0.79–2.57
North West	177	2.37**	1.37–4.12	2.73**	1.58–4.72
South East	185	1.18	0.71–1.95	1.13	0.55–1.95
South South	165	1.52**	0.89–2.58	1.55	0.83–2.72
South West	387	1.65**	1.03–2.65	1.69**	1.03–2.76
Wealth Index					
Poorest (RC)	51	1		1	
Poorer	108	0.81	0.38–1.70	0.62	0.24–1.58
Middle	178	1.17	0.62–2.20	0.81	0.35–1.87
Richer	330	1.20	0.65–2.22	0.81	0.35–1.86
Richest	532	1.03	0.56–1.89	0.69	0.29–1.66
Education					
No education (RC)	140	1		1	
Primary	165	1.36	0.77–2.40	1.51	0.82–2.77
Secondary	620	1.31	0.82–2.09	2.00**	1.16–3.43
Tertiary	274	1.15	0.69–1.92	1.97**	1.09–3.58
Age					
15–19 (RC)	8	1		1	
20–24	92	7.14	0.80–63.64	5.80	0.59–56.99
25–29	325	5.36	0.64–45.01	3.86	0.42–35.89
30–34	334	6.44	0.76–54.87	4.35	0.45–41.88
35–39	277	8.56**	1.01–72.64	5.04	0.52–48.78
40–44	122	15.07**	1.74–130.45	6.42	0.64–64.03
45–49	41	15.36**	1.63–145.22	5.95	0.53–66.83

^*^ *p* < 0.001, **p < 0.05, *RC* Reference Category, *OR* Odds Ratio, *CI* Confidence Interval, *aOR* Adjusted Odds Ratio

In the multivariable results, the adjusted odds ratio results in Table 2 show that women who visited a health facility in the last 12 months before the survey were 4% (aOR = 0.6, CI: 0.4—0.8) less likely to discontinue modern contraceptive methods than women who have not visited a health facility in the last 12 months before the survey. Women whose marital duration was 20 years and above were 3 times (aOR = 3.0, CI: 1.5—6.2) more likely to discontinue modern contraceptive methods than women whose marital duration was 0–4 years. Also, women from the North West region of the country were found to be about 3 times (aOR = 2.7, CI: 1.6—4.7) more likely to discontinue modern contraceptive methods than women from the North Central. Likewise, women in South West region were 70% (aOR = 1.7, CI: 1.0—2.8) more likely to discontinue modern contraceptive methods than their counterparts from the North Central. As regards the level of education, the data showed that women with secondary education and women with tertiary education were 2 times (aOR = 2.0, CI: 1.2 – 3.6; aOR = 2.0, CI: 1.09 – 3.58) respectively more likely to discontinue modern contraceptive methods than their counterparts with no education. Age, wealth quintile and media exposure did not appear to be significantly associated with modern contraceptive methods discontinuation.

## Discussion

This study examined the prevalence and associated factors of modern contraceptive discontinuation. We sought to provide a better understanding of related factors influencing modern contraceptive discontinuation in Nigeria. The study found a significant number of women who discontinued using contraceptives while at risk of pregnancy. Previous studies have also reported this finding of contraceptive discontinuation in the 12 months among women at risk of pregnancy [5, 19]. This, however, has serious negative reproductive health consequences. This is because contraceptive discontinuation for reasons other than the desire for pregnancy is associated with high rates of unintended pregnancies leading to unsafe abortions, maternal morbidity and mortality in Nigeria [25]. This pattern of results (high rate of contraceptive discontinuation for women at risk of pregnancy) is unexpected and challenging because a substantial proportion of women discontinued contraception for purposes beyond the desire to be pregnant. Other plausible reasons found in this study responsible for a high rate of contraceptive discontinuation included infrequent sex, inconvenient use, ineffective methods, methods failure, and side effects, among others, which were established in this study.

Just as established by mounting evidence in scholarly literature that a higher proportion of women are short–term contraceptive users globally, our study found that short-term contraceptive methods (injectable, pills and condoms) are the most discontinued modern contraceptive method. This finding corroborated existing findings [5]. The observable fact attributed to this is that it is easier to discontinue short-term contraception without professional service of medical/health providers unlike the Implant and IUD, or other long-acting reversible contraceptives (LARC). This might have accounted for the reason why women discontinued short-term contraceptive methods more often.

 This study found that the intention to become pregnant is the commonest reason for modern contraceptive discontinuation. This finding aligns with what one would expect of women of reproductive age who wanted to be pregnant [17, 19, 21, 29]. However, side effects/health concerns and contraceptive failure were also mentioned as reasons why women discontinued modern contraceptive methods. This is consistent with previous studies [6, 29, 30] and lends credence to the need for adequate and accurate information and counselling on the failure and side effects of modern contraceptives. 

Furthermore, our study established some significant correlates of modern contraceptive discontinuation among women aged 15–49. These include; a visit to a health facility in the 12 months before the survey, marital duration, education, and region of residence. The study found low discontinuation among women who visited a health facility in the last 12 months before the survey. This is consistent with a previous study [29]. The probable explanation for this is that women who visited health facilities in the reference period have a higher tendency to adhere to counselling and information on the benefits of using contraceptive methods as this could be easily assessed in the health facilities thus, reducing discontinuation. Even with other proximate barriers that drive contraceptive discontinuation, health facility visitation is likely the single most important variable that reduces contraceptive discontinuation among women at risk. The instructive message for policymakers is to increase both health facility and community-based family planning outreach and/or integrate family planning programmes in maternal health care services.

The study also found that marital duration influenced modern contraceptive discontinuation, especially short-term contraceptive methods. Women with longer marital duration may have discontinued contraception than their counterparts who are younger in marriage. Other studies [6, 17, 29] have indicated that a longer duration of marriage may affect contraceptive use. This finding may be attributable to a reduced need for contraception by women who no longer desire to have more children or are older in marriage thereby using a long-acting and reversible contraceptive (LARC) method rather than short-term contraceptive methods. In like manner, married women with long years in marriage may have reached menopause or be less fertile. Furthermore, findings showed that women living in the North West had higher odds of modern contraceptive discontinuation. This finding corroborates existing findings [6, 29]. Though there is high contraceptive discontinuation across all the regions as it is nationally, a higher rate in North-West, for example, could be because of low knowledge of family planning, the predominant effect of Islamic pronatalist and religious doctrines. This however has public health implications associated with a higher chance of mistimed and unwanted pregnancies, unwanted births and unsafe abortions leading to increasing risks of maternal morbidity and mortality in Nigeria. Therefore, governments, stakeholders and health care providers should increase community sensitisation and counselling about contraceptives side effect and encourage women on the use of long-acting and reversible methods which cannot be changed abruptly without the service of health providers. Also, they should design and implement regional-specific policies to reduce contraceptive discontinuation, especially among women at risk in Nigeria.

### Strengths and limitations of the study

The data used for the study is quantitative and provides reliable nationally representative estimates that are generalizable for modern contraceptive discontinuation in Nigeria. The study also departs from previous studies as it considered the calendar year episodes of contraception history. Thus, as a consequence, the result is robust and can be used as evidence for policy formulation and design of family planning programmes for improved contraceptive use in the context of Nigeria and similar settings. However, it should be noted that the survey design is cross-sectional. Thus, causal inferences cannot be established. In addition to this, there is a possibility of recall bias due to the retrospective nature of the study. Finally, other important community and individual associated factors previously established in some studies were left out because they were either not available or missing in the dataset.

## Conclusion

The study found a high number of women who discontinued modern contraception while still at risk of becoming pregnant. Associated factors of modern contraception discontinuation were women's visitation to a health facility in the last 12 months of the observation period, marital duration, education, and region of residence. To increase modern contraceptive use in Nigeria and discourage discontinuation, it is imperative for health care providers to address the discontinuation of contraception through counselling, particularly among short-term users as well as strengthen the use of contraception among those who are still at risk of becoming pregnant. Governments and stakeholders should employ strategies to prevent discontinuation by dissuading users from seeing discontinuation as an option while still at risk of having pregnancy owing to the side effect of contraceptive methods.

## Data Availability

The datasets are available in the Nigeria Demographic and Health repository, http://dhsprogram.com/data/available-datasets.cfm. Permission to use the data was obtained at the Measure DHS website www.measuredhs.com upon request.

## Abbreviations

AOR: Adjusted Odds Ratio
CI: Confidence Interval
DHS: Demographic and Health Survey
ICF: ICF International
IRB: Institutional Review Board
IUD: FP: Family Planning
NDHS: Nigeria Demographic and Health Survey
NPC: National Population Commission
OR: Odds Ratio
SDG: Sustainable Development Goals, WHO: World Health Organization
UNFPA: United Nations Population Fund
UNICEF: United Nations Children’s Fund
